# Interferon-Free Regimens and Direct-Acting Antiviral Agents for Delta Hepatitis: Are We There Yet?

**DOI:** 10.3390/cimb45100498

**Published:** 2023-09-28

**Authors:** Roxana Nemteanu, Andreea Clim, Corina Elena Hincu, Liliana Gheorghe, Irina Ciortescu, Alina Plesa

**Affiliations:** 1 Medical I Department, Grigore T. Popa University of Medicine and Pharmacy, 700100 Iasi, Romania; andreea.clim13@yahoo.com (A.C.); lgheorghe123@gmail.com (L.G.); alinaplesaro@yahoo.com (A.P.); 2 Institute of Gastroenterology and Hepatology, “Sfantul. Spiridon” University Hospital, 700111 Iasi, Romania; 3 Department of Radiology, “Sfantul Spiridon” Hospital, 700111 Iasi, Romania; corinaelenahincu@gmail.com

**Keywords:** delta hepatitis, direct antiviral treatment, pathogenic mechanisms

## Abstract

Chronic delta hepatitis is a global health problem. Although a smaller percentage of chronic HBV-infected patients are coinfected with the hepatitis delta virus, these patients have a higher risk of an accelerated progression to fulminant “delta hepatitis”, cirrhosis, hepatic decompensation, and hepatocellular carcinoma, putting a financial strain on the healthcare system and increasing the need for a liver transplant. Since its discovery, tremendous efforts have been directed toward understanding the intricate pathogenic mechanisms, discovering the complex viral replication process, the essential replicative intermediates, and cell division-mediated viral spread, which enables virion viability. The consideration of the interaction between HBV and HDV is crucial in the process of developing novel pharmaceuticals. Until just recently, interferon-based therapy was the only treatment available worldwide. This review aims to present the recent advancements in understanding the life cycle of HDV, which have consequently facilitated the development of innovative drug classes. Additionally, we will examine the antiviral strategies currently in phases II and III of development, including bulevirtide (an entry inhibitor), lonafarnib (a prenylation inhibitor), and REP 2139 (an HBsAg release inhibitor).

## 1. Introduction

The World Health Organization (WHO) reported that chronic viral B and C hepatitis are major global public health threats, with an estimated 354 million people living with chronic hepatitis B (296 million) and C (58 million), accounting for over 1.1 million deaths in 2019, mainly due to the complications of cirrhosis and hepatocellular cancer [1]. In addition, recent data reporting an alarming prevalence of hepatitis delta virus (HDV) coinfection, ranging from 12 million up to 60 million individuals, have surfaced, with an estimated 10% of hepatitis B virus (HBV) surface antigen (HBsAg)-positive patients [2,3]. These statistics are indeed disturbing, but optimistic perspectives on emerging new drugs for these silent enemies are foreseen on the horizon. The WHO aims to eliminate viral hepatitis as a public health threat by 2030, defined as a 90% reduction in new chronic infections and a 65% reduction in mortality [1]. The feasibility of achieving global eradication is supported by the distinctive attributes of these viruses, the existence of reliable diagnostic techniques, and the availability of cost-effective or cost-saving interventions. Nevertheless, achieving the global eradication of HBV and HCV by the year 2030 is an ambitious objective that necessitates substantial efforts and extensive work throughout the forthcoming decade. To achieve the objective set forth by the WHO in eradicating viral hepatitis, it is imperative to enhance the global diagnostic coverage from 9–20% in 2015 to 90% by the year 2030. Furthermore, it is imperative to enhance the extent of treatment coverage, aiming to increase it from 7–8% in 2015 to 80% by the year 2030 [1,2].

HDV was first described in Torino by Rizzetto and colleagues in the mid-1970s [4]. HDV was initially considered a new HBV antigen, but shortly after, its unique viral structure was identified and classified as a defective satellite virus of HBV [5]. Currently, substantial efforts are being made by the scientific community to reduce the burden of chronic liver disease by actively screening populations during micro-elimination campaigns and providing linkage to care for exposed patients [1,3]. Although a smaller percentage of chronic HBV-infected patients are coinfected with HDV, these patients have a higher risk of an accelerated progression to fulminant “delta hepatitis”, cirrhosis, hepatic decompensation, and hepatocellular carcinoma, putting a financial strain on the healthcare system and increasing the need for liver transplantation [5,6]. Therefore, physicians should be aware of and actively search for this virologic association to enable expeditious access to antiviral treatment.

In the current manuscript, we aim to review the recent scientific advances regarding the life cycle, the pathogenic potential of HDV, and the development of several new treatment options for patients with HBV and HDV co-infection.

## 2. Epidemiology of Hepatitis Delta Virus Genotypes

The geographic distribution of HDV infection is quite heterogeneous. Since 1986, eight distinct genotypes have been identified (from 1 to 8), with the variability of nucleotide sequencing as high as 35% and a ubiquitous geographic distribution [7,8]. The Type 1 genome has a global dispersion, most frequently encountered in North America, the Middle East, and Europe. In addition, genotypes-5, -6, -7, and -8 have also been identified in different regions of Africa, while types 7 and 8 have been commonly found in Cameroon and Gabon [9]. The type 2 genotype is frequent in Asia and Russia [8,10]. Among European countries, type 1 was the most commonly found genotype. For example, in European countries such as Romania, Italy, and Spain, only the type 1 genotype was identified [11,12]. Genotype recognition may be useful as these viral subtypes are associated with distinct disease patterns. For example, type 2 is associated with a less aggressive form of the disease compared to type 1, and partial explanations of this virulence reside in the packing signal (the 19-aminoacid extension of the L-HDAg) [11]. Genotype 4 (the old genotype IIb) occurs in Japan and Taiwan and usually leads to milder forms of liver dysfunction, yet the genotype-4 isolate from Okinawa is associated with a faster progression to advanced liver fibrosis [13]. HDV-genotypes 5 to 8 are found in patients from Africa who migrated to Northern Europe and have a less well-characterized natural history of the infection [14]. Genotype recognition may therefore be useful in future endeavors to design more specific and targeted antiviral medications.

## 3. Hepatitis Delta Virus Structure, Replication, and Pathogenic Mechanisms

Compared to other viruses causing hepatitis, HDV is unique in that it needs the presence and helper function of HBV to infect hepatocytes [15]. Structurally, HDV is a spherical, 35 to 41 nm in diameter, enveloped transmissible virus belonging to the Deltaviridae family, genus Deltavirus [16]. In 1986, genomic sequencing showed that HDV contains a circular RNA molecule of negative polarity comprising close to 1700 nucleotides with no nucleocapsid structure, making HDV the smallest known viral pathogen in humans [17,18]. It shares common features with plant viroids and virusoids in its replication mechanism, which uses RNA polymerase and can self-cleave the circular RNA into a linear pattern [19]. The HDV ribozyme is the fastest naturally occurring self-cleaving RNA [20]. Essentially, the HDV virion has a ribonucleoprotein (RNP) complex inside and an HBV-derived envelope outside. Its uniqueness extends beyond its structural complexities to an ongoing dynamic interaction between the infected cells and viral strains [21]. Numerous follow-up studies have shown that viral load may longitudinally vary and that patients co-infected with HDV express lower HBV viral loads compared to non-infected individuals, but the exact mechanism is still unknown [22].

When the host’s transcriptional machinery is compromised, HDV replicates its RNA genome through a rolling circle method that is unknown to animal cells, as described by Rizzetto et al. in their research on the virus’ unusual replication pattern [23]. There is an obvious dependency of HDV on HBV exclusively due to the evolutionary adaptation of HDV in using HBV-encoded surface antigen HBsAg for its release and de novo infection [24]. Patients may be diagnosed with an HDV infection either simultaneously with an HBV infection or as a superinfection in patients already chronically infected with HBV [25]. The HBV envelope proteins can be obtained from HBV genomes present in cell nuclei (covalently closed circular DNA, cccDNA) and also from chromosomally integrated sequences, equally contributing to HDV release [23,24,25].

**Figure 1:**
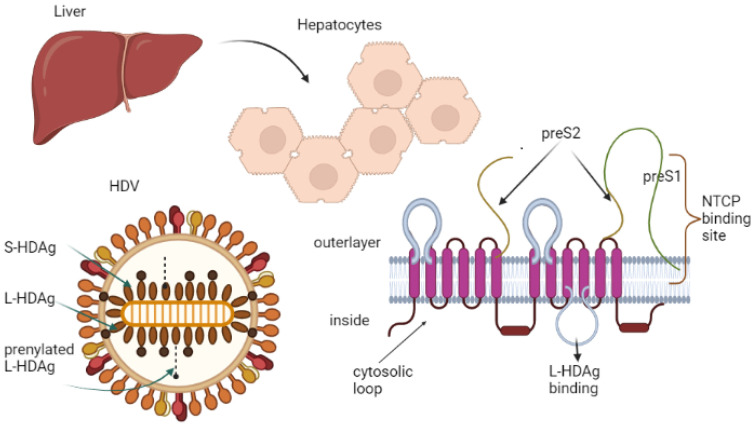
Schematic representation of HDV virion and envelope proteins (L-HDAg, S-HDAg the large and small isoforms of the hepatitis delta antigen, NTCP sodium taurocholate co-transporting polypeptide).

For hepatocytes to interact with HBV and HDV, a specific connection between the binding domain in the preS-1-part of the HBV L-protein and the hepatic sodium taurocholate co-transporting polypeptide (NTCP) receptor has to materialize [26]. The NTCP is exclusively found at the basolateral membrane of differentiated hepatocytes and acts as a mediator of bile acid transport. However, some studies report that the down-regulation of NTCP in proliferating hepatocytes may be the reason that the regenerative hepatocytes are protected from reinfection or de novo infection, a hypothesis worth further investigating [26,27]. NTCP expression is sufficient to enable HDV cell entry and the onset of the replication process, but virion release does not occur when HBV envelope proteins are not present [27]. This can be surpassed by cells that express both the NTCP receptor and the HBV envelope proteins (Figure 1). Interestingly, proliferating hepatocytes transformed into hepatoma cells and possibly tumor cells lack NTCP25 and do not support entry of HBV and HDV, an intriguing angle worth exploring in future clinical trials to develop targeted drugs [28].

After infecting the hepatocytes through the NTCP, HDV replicates in the nucleus using the host RNA polymerase II and a double rolling-circle amplification process [29]. The RNA polymerase II enzyme is normally a DNA-dependent enzyme that is hijacked by HDV to use its RNA as a stencil. Such ‘molecular mimicry’ complexes of dsDNA allow the host DNA-dependent RNA polymerase II to accomplish RNA-dependent RNA synthesis [30]. Evidence has shown that HDAg functionally interacts with the clamp of polymerase II, a mobile structure that fixes DNA and RNA in place through transcription, utilizing reconstituted elongation complexes and site-specific photo-crosslinking. Surprisingly, HDAg influences which nucleotide is integrated in addition to speeding up the pace of elongation. These results, together with those from previous research, suggested a paradigm in which HDAg interacts with the clamp, loosens it, and speeds up the forward translocation of polymerase II at the expense of fidelity [31]. By lowering transcriptional fidelity in terms of both template recognition and the discrimination of incoming nucleotides, HDAg may support polymerase II’s novel RNA-dependent RNA synthesis [32].

The double rolling-circle amplification process is complex and shows the lengths to which this small virus will go to replicate. The rolling process begins with the synthesis of multimeric linear transcripts from the genomic template. The linear product, which has undergone autocatalytic cleavage and then ligation by the ribozyme and host ligases, is finally delivered as the neo-synthesized circular antigenomic RNA [33]. The antigenomic RNA serves as a pattern for the replication of the circular genomic RNA by a similar process [34]. HDAg is the only protein expressed by the HDV. Following post-translational alteration, two additional RNAs can be identified: the antigenomic (AG) HDV RNA and a smaller mRNA coding for the two isoforms of the hepatitis delta antigen (large: L-HDAg and small: S-HDAg). Both antigens bind to genomic RNA, and they share different roles during the replication process. The replication process is also influenced by the activity of adenosine deaminase acting on RNA 1 (ADAR1) [35,36]. The role of the enzyme is to edit the HDV RNA as a means to produce the two small and large versions of HDAg. The S-HDAg increases genome replication and dominates the early stages of cell infection, while the L-HDAg promotes virus particle formation with increased activity in the later stages. The L-HDAg connects the RNP to the HBV envelope via a binding domain composed of 19 C-terminal amino acids. It also negatively regulates replication and triggers the envelopment of the virus into the HBV surface proteins [24,25]. All these steps are mandatory for creating delta virions.

Critical to virion assembly is the prenylation of the L-HDAg, which inhibits replication and drives the HDV RNA to combine with the HBsAg to assemble the virion. The integrity of surface proteins (HBsAg) is also extremely important. The envelope contains three HBV envelope proteins: small-HBsAg (S-HBsAg), medium-HBsAg (M-HBsAg), and large-HBsAg (L-HBsAg) [37]. They share the same structure, with additional N-terminal extensions for the medium (preS2) and the large (preS1, preS2). All surface structures need to be intact, especially the L-preS1 domain, which is critical for binding and cell entry. Furthermore, the prenylation of L-HDAg is essential for envelope acquisition. The RNP is released and then transported to the nucleus to start RNA replication after membrane fusion. The generated genome serves as the blueprint for the initial rolling circle amplification. To control viral replication or bind to HDV RNA to generate RNP, both intact and prenylated S-HDAg and L-HDAg are delivered into the nucleus. By interacting with L-HDAg and S-HBsAg, the genome-containing RNP may subsequently be transported to the cytoplasm and incorporated into the HBV envelope. The endoplasmic reticulum-Golgi secretory route is then employed to release HDV virions [7,22].

## 4. The Role of Interferon in Modulating Disease Behavior and Its Use in Therapy

In normal circumstances, a viral threat activates the body’s innate immune system, which aims to suppress the antigenic attack by triggering direct antiviral responses via interferon (IFN) and modulating the induction of adaptive immune responses [38]. The accuracy, tempo, and strength dictate the outcome of infection by either abolishing it or allowing the persistence of infective antigens. The viral antigens are recognized by the immune cells (more specifically by sensitive receptors such as toll-like receptors) as pathogen-associated molecular patterns, and an innate immune response is then initiated [39]. This immune response is a combination of signaling events that allow the production of different types of interferon. As a consequence of increased IFN production and binding to their corresponding IFN receptors situated on both infected and non-infected cells, a cascade of molecular events is then triggered. This in return activates kinases such as Janus kinases 1/2 and tyrosine kinase exerting direct and indirect antiviral activities via IFN-stimulated genes [40].

In HDV-infected cell lines, primary human hepatocytes, and animal models, innate immune responses to HDV have been observed. Experimental trials have reported that HBV/HDV co-infection activates IFNs and human cytokines in a humanized mouse model repopulated with primary human hepatocytes, while HBV mono-infection remains silent and does not trigger the IFN response of hepatocytes or interfere with the innate immune-sensing functions of hepatocytes. Although there is compelling evidence that HDV replication induces IFN response, HDV is a relatively moderate stimulator compared to some other RNA viruses [41]. HDV is considered to interfere with interferon IFNα signaling by interrupting the activation and migration of STAT proteins, inducing the persistence of HDV and impairing IFN-based therapies [42]. The weak adaptative immune response is also responsible for chronic exposure and progression toward liver cirrhosis.

### The Use of IFNs and Therapy Outcome

Interferon-based therapeutic regimes (standard INFα or pegylated pegIFNα, monotherapy, or in combination with ribavirin) have been used since the 1980s, and are still being recommended for managing patients with HBV and HDV, with significant side effects and suboptimal responses for patients. The pegIFNα is better tolerated compared to INFα, showing better compliance scores (weekly subcutaneous injections), fewer side effects, and overall superior results of sustained virological suppression response [43]. The exact mechanism by which patients show undetectable viremia remains unknown. It is suspected that IFN can suppress infected cell division and mediate the spread of cells containing delta viromes, leading to a reduction in HDV replication and dissemination [35,39]. However, total HDV clearance obtained using IFNα monotherapy is suboptimal, which is why its clinical use has been significantly overpowered by a more patient-friendly version of peg-IFNα. Although the rate of HDV complete elimination is low, IFN manages to decrease viral load in the majority of patients [39]. Nevertheless, more recent data on the efficacy of pegIFN-2a and -2b show disappointing results, as reported by Abrdakham et al. The authors concluded that pegIFNα-2a or -2b has limited efficacy in treating HDV patients because only one-third of chronic HDV patients achieved clearance and normalized liver enzymes. In addition, HBsAg clearance with seroconversion to anti-HBs was achieved in 1% of patients [44]. The normalization of ALT levels is also an indicator of viral response, and the elevation of ALT levels correlated with HDV RNA seropositivity at year 1 among patients with HBV treated with nucleotide/nucleoside analogs or hepatocellular occurrence, as reported by Jang et al. [45]. To date, there is no pharmacological therapy that can cure HDV infection [7]. However, the recent advances in understanding disease pathophysiological mechanisms have allowed the development of new drugs by addressing intricate molecular pathways. Therefore, currently, a few promising drugs are in phases 2 and 3 of clinical trials for the treatment of HDV and HBV co-infection.

## 5. Emerging New Drugs: Targeted Molecular Therapy

**Figure 2:**
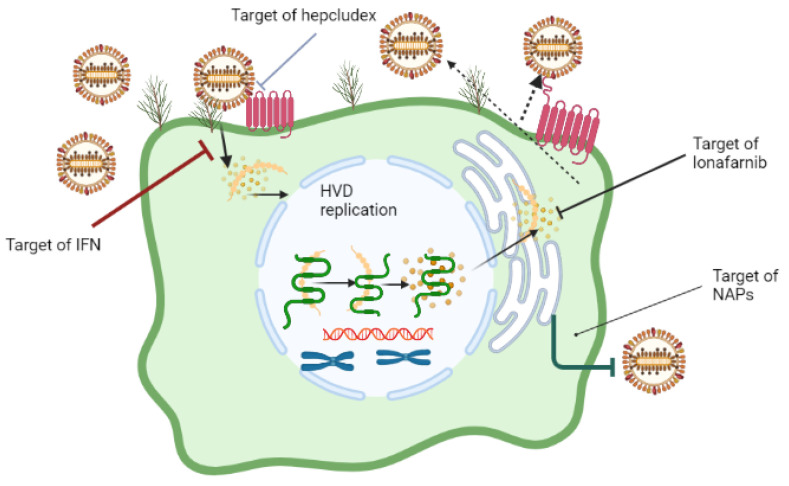
HDV spreading pathways and the targeted site of direct antivirals. The coinfection of naive hepatocytes with HBV and HDV results in the production of progeny HD virions. Similarly, the infection of hepatocytes that carry integrated HDV DNA with HDV also leads to the production of progeny HD virions. The progeny HD virions have the ability to infect adjacent undamaged hepatocytes or super-infect hepatocytes that are already positive for HBV. Bulevirtide and lonafarnib have been shown to effectively inhibit the extracellular spreading pathway by acting as an entry inhibitor and a secretion inhibitor, respectively. Bulevirtide (Hepcludex) is an entry inhibitor, Lonafarnib acts as an oral prenylation inhibitor, and Nucleic acid polymers block viral entry.

The unique and peculiar pathobiology of HDV has been a contributing factor to the suboptimal response to available therapeutic options [46]. Entry inhibitors, farnesyl transferase inhibitors, and nucleic acid polymers have been proposed as novel drug options for HBV/HDV treatment in clinical trials (Figure 2). Carbamoyl-phosphate synthetase 2, aspartate transcarbamylase, and dihydroorotase have been identified as proteins that are important for HDV replication [47,48]. In addition, pevonedistat and N-(phosphonoacetyl)-L-aspartic acid could impede HDV replication and HBV transcription [49].

The effectiveness of HDV egress and entry, and consequently the ability of lonafarnib or bulevirtide to block assembly or entry, are both heavily influenced by variations in HDV genomes and HBV envelope proteins. The pathogenicity of HDV, immunological reactions, and the effectiveness of innovative medication regimens may all be impacted by these variations [24].

Although interferon treatment has some downsides, there are a few aspects worth discussing. Firstly, IFN receptors are expressed by the majority of cells, explaining the significant side effects that limit their use. However, a favorable response is detected among some patients with co-infection with HDV, which is why the use of interferon has been reconsidered. More specifically, interferon lambda (IFNλ), a type III IFN, has receptors restricted to different cell types including hepatocytes and epithelial cells (found in skin, the intestine, and lungs) compared to IFNα/β. Therefore, typical side effects of IFN treatment are not expected while using IFNλ and its use has already been assessed during clinical trials [16,44]. For example, in the phase II clinical trial called LIMT designed by Hamid et al. including 30 patients with HBV and HDV treated with pegIFNλ in monotherapy (120 μg) or in combination with tenofovir or entecavir (180 μg pegIFNλ), while evaluating the safety profile, tolerability, and efficacy, the authors found (at week 72) better outcomes in the tenofovir combination group [50]. In another phase II open-label clinical trial, patients treated with peg-IFNλ 180 μg, ritonavir, and lonafarnib for 24 weeks showed a good response at 24 weeks off-therapy defined by a reduction in HDV RNA < 40 IU/mL in 19% of cases. Treatment with pegIFNλ was associated with better or comparable rates of virologic response with fewer extrahepatic adverse events than IFNα [51].

### 5.1. New Generation Drugs: Bulevirtide

On the opposite end of the spectrum, interferon-free alternatives, such as Bulevirtide, presently referred to as Hepcludex (formerly known as Myrcludex B), are now available. Bulevirtide is a synthetic peptide consisting of 47 amino acids, specifically designed to function as an inhibitor of the NTCP, the receptor of HDV/HBV inhibiting virus entry [52].

Several safety and efficacy clinical trials have already released their results, comparing different therapeutic regimens either in monotherapy with bulevirtide or combined therapy with pegIFNα or TDF.

Wedemeyer H et al. conducted a clinical trial (Myr202) involving 120 patients with chronic liver disease who were being treated with TDF. These patients were randomly assigned to receive varying doses of bulevirtide (2, 5, or 10 mg/day) or TDF as a monotherapy (245 mg/day) for a duration of 24 weeks. The study observed that a significant proportion of patients (77%) who received a 10 mg dose of bulevirtide achieved a 2-log reduction or undetectable levels of HDV RNA at week 24. In contrast, only a small percentage (3%) of patients treated alone with TDF accomplished the primary goal [53]. The Myr-203 extension trial involved the randomization of ninety patients who were co-infected with HBV and HDV into six treatment arms. The study successfully demonstrated that 53.3% of patients who received a combination therapy of 2 mg bulevirtide and pegIFNα achieved the primary endpoint of having HDV RNA levels below the lower limit of detection of 10 IU/mL at week 72 (24 weeks after discontinuing medication). The achievement of normal ALT levels and the absence of HBsAg, or a reduction of more than 1 log IU/mL, were exclusively observed in patients who had combination therapy with bulevirtide and pegIFNα [54]. The multicenter, randomized phase 3 clinical study Myr-301 reported that bulevirtide proved to be a safe and well-tolerated drug over the 48-week timeframe [55].

Currently, the 2 mg dose of bulevirtide is the only European Medicines Agency-approved drug for HDV treatment [56]. Based on preliminary data, 3 strategies could be envisioned. A rigorous assessment of the disease stage and fibrosis grade should be performed, in conjunction with an estimation of contraindications for pegIFNα use. Moderate to severe chronic liver disease or compensated cirrhosis without oesophageal/gastric varices with no contraindication to IFN, bulevirtide 2 mg + pegIFNα for 48 weeks or pegIFNα monotherapy for 48 weeks can be administered. In case of contraindications to IFN, bulevirtide 2 mg in monotherapy can be prescribed. In the case of compensated cirrhosis with oesophageal/gastric varices, 2 mg of bulevirtide in monotherapy is safe and effective. For decompensated liver cirrhosis, no approved therapy is currently available [56].

### 5.2. Lonafarnib

Remarkably, lonafarnib, a pharmaceutical compound utilized in the therapeutic intervention of cancer and progeria, has demonstrated significant potential in effectively managing individuals afflicted with HDV. It functions by inhibiting farnesyl transferase, hence disrupting many cellular regulatory pathways. This inhibition prevents the prenylation of L-HDAg and subsequently reduces its interaction with HBsAg. The antiviral mechanism inhibits the viral life cycle during the assembly phase, while also impeding the secretion of HDV virions.

The drug is currently undergoing clinical trials, and in subsequent studies, LOWER-2 or *LIMT,* lower doses of lonafarnib were administered in association with ritonavir and pegIFNλ, without a negative impact on the antiviral response [57]. Lonafarnib use has shown a decrease in viral load in treated patients, but with discouraging side effects such as diarrhea, nausea, bloating, and weight loss, which were managed by a reduction in lonafarnib doses boosted with ritonavir or pegIFNα [58]. The first single-center, phase 2 pilot study called LOWR HDV-1 (LOnafarnib With and without Ritonavir in HDV—1) was designed as a seven-arm, parallel, open-label clinical trial to dose twenty-one patients across seven groups (three patients per group). The patients were orally treated with different doses of lonafarnib, in association with ritonavir or pegIFNα for a duration of 8 to 12 weeks. The study aimed to see whether higher or more frequent dosing of lonafarnib would be more efficacious and if extending the treatment duration to 12 weeks would lead to further HDV RNA declines than previously observed with 4 weeks of dosing. The authors concluded that combining lonafarnib with pegIFNα achieved more substantial and rapid HDV RNA reduction and that ritonavir boosting along with lowering lonafarnib doses does not hinder the expected antiviral responses [59].

In their latest study on dose-finding, Yurdaydin et al. sought to determine the most effective and well-tolerated combination regimens for the longer-term administration of lonafarnib and ritonavir, with or without IFNα. The LOWR-2 trial, which was conducted in an open-label manner, included a total of 55 participants who were diagnosed with chronic HDV. The authors reported that the inclusion of pegIFN increases the efficacy of the promising all-oral medication lonafarnib when used in combination with low-dose ritonavir [60]. The safety and efficacy of lonafarnib were further assessed in two important studies, LOWR HDV-3 and LOWR HDV-4. The studies documented the decrease in HDV RNA levels in patients receiving a combination of lonafarnib with ritonavir, and the response to rapid step-wise increases of lonafarnib to high doses, respectively, with overall optimistic results [61,62]

### 5.3. Nucleic Acid Polymers

Nucleic acid polymers (NAPs) are phosphorothioated oligonucleotides that inhibit the assembly and release of HBsAg-coated viral particles. Their exact mechanism of action is incompletely understood, but they act as amphipathic molecules. They achieve this feature by forming cooperative hydrophobic connections with a target helix’s hydrophobic surface, which prevents the target from changing conformation or interacting with other amphipathic helixes. The initial REP2055 was not well tolerated, so REP 2139 replaced REP2055 [63].

REP 2139 is the first NAP studied in clinical trials for HDV treatment. Bazinet et al., in an open-label, non-randomized, phase 2 clinical trial, assessed the safety and efficacy of REP 2139 and pegIFNα in 12 naive patients with chronic delta hepatitis. All patients received 500 mg iv REP 2139 once per week for 15 weeks, followed by combined therapy with 250 mg intravenous REP 2139 and 180 μg pegIFNα once per week for 15 weeks, then monotherapy with pegIFNα 180 μg once per week for 33 weeks. The combination therapy proved safe for and well tolerated by patients, with effective inhibition of HDV and sustained remission after a one-year follow-up. Interestingly, the anti-HDV action of NAPs was found to be pangenomic in relation to HBV envelope proteins, and it was not rendered ineffective by HBsAg alterations that are typically linked with immune escape in HBV. In addition, the observation that NAPs have the ability to inhibit HDV at the point of viral entry implies their potential efficacy in managing the dissemination of HDV within a liver that is chronically infected with HBV. However, although these results are promising, there is still a lack of information regarding the safety profile of the long-term use of NAPs, and future clinical trials should answer more stringent questions [64]. Further research on new-generation drugs employed as combination therapy for HDV treatment is displayed in Table 1.

Despite the evident therapeutic effectiveness of bulevirtide and lonafarnib, which are direct-acting antivirals, a resurgence of HDV was unfortunately noted after discontinuing treatment. The efficacy of IFNs as a monotherapy was unsatisfactory, as previously reported [44]. In addition, the efficacy of either drug in eliminating HDV as a monotherapy remains uncertain. In order to accomplish a sustained viral response HDV, it will be necessary to implement a comprehensive and sustained treatment strategy that includes the complete inhibition of specific stages in the replication process. Recent clinical studies have shown that the combination of lonafarnib, bulevirtide, and REP-2139 with peg-IFNα exhibited additive or, in the case of bulevirtide and lonafarnib, even stronger synergistic antiviral effects [50,51,52,53]. These effects are characterized by faster and more significant reductions in serum HDV RNA, higher rates of off-treatment responses, and lower rates of relapse. Therefore, monotherapy with either direct-acting antivirals or IFNs proved ineffective, yet combined therapy addressing more links in the replicative process and reduction of viral spread with IFNs and direct-acting antivirals has shown promising clinical results [49]. Therefore, the future goal will be to design the perfect combination of therapies in order to cure HDV infection. Future research should also clarify whether a cure for HDV may be attained without the removal of hepatocytes that carry replicating HBV or encode HBsAg from integrated forms in the liver [52]. Given that the latter objective is still a considerable distance away, the urgent requirement is directed toward designing drugs that can effectively manage HDV and restore normal liver function in these patients.

## 6. Future Directions

Four decades into HDV diagnosis, the first anti-HDV therapeutic regime has been approved in Europe, offering patients an optimistic perspective on a possible cure for HDV [65]. Combinations of direct antivirals with pegIFNs accelerate the reduction of viral loads by synergistically addressing both pathways of persistence. As the scientific community uncovers more information related to the HDV life cycle and pathogenetic mechanisms, this knowledge will change the treatment dynamic. Cellular kinases, vaccines, estrogen receptor inhibitors, and gene therapy are currently under assessment as new therapeutic tools to fight against HDV [47,48]. The presence of eight HDV genotypes and the viral RNA ribozyme explain the extreme genetic variability of the HDV genome and the selection of resistant variants [11]. In conclusion, although the therapy for chronic delta hepatitis is still in its infancy, it is envisioned that soon innovative direct-acting medications will become available to cure HDV infection.

## Figures and Tables

**Table 1:** Clinical trials assessing the safety, efficacy, and treatment response to new antiviral regimens.

	Name of Agent/Agents Used	Number of Participants	Study Type	Phase of Clinical Trial/Trial ID	Summary of Clinical Trial Outcomes
Lonafarnib Oral prenylation inhibitor	Lonafarnib Placebo	14	Randomized Interventional	Phase 2/ NCT01495585	– safety and effectiveness of lonafarnib
	Lonafarnib Ritonavir Interferon λ	26	Interventional	Phase 2/ NCT03600714	– safety and treatment response in managing patients with HDV-treated Lonafarnib, Ritonavir, and IFN λ – primary outcome: the decline of HDV RNA viral titer of >2 logs from baseline (24 weeks of therapy), – secondary outcomes: reduction in histologic inflammatory scores at week 24 post-treatment follow-up, normalization of serum ALT, and loss of HBsAg.
	Lonafarnib Ritonavir Placebo	22	Interventional	Phase 2/ NCT02511431	– assesses the decline of HDV RNA of >2 Logs from baseline at 12 weeks of treatment.
Bulevirtide Entry inhibitor	Bulevirtide pegIFN α-2a Tenofovir	90	Multicenter, Open-label, Randomized, Comparative, parallel-arm	Phase 2/ NCT02888106	– explore the efficacy of treatment with bulevirtide used as a monotherapy and in combination with peg-IFNα and Tenofovir compared to monotherapy with pegIFNα in patients with chronic HBV-HDV co-infection, based on the achievement of undetectable viral load at the end of the follow-up period 6 months (24 weeks) after the end of treatment. – undetectable HDV RNA by PCR at Week 72 (end of the follow-up period).
	Bulevirtide Tenofovir	120	Multicenter, Open-label, Randomized Interventional	Phase 2/ NCT03546621	– efficacy and safety of three doses of bulevirtide for 24 weeks in combination with Tenofovir compared to Tenofovir alone to suppress HBV replication in patients with HDV at week 24.
	Bulevirtide pegIFNα	175	Randomized Interventional Multicenter, Open-label	Phase 2b/ NCT03852433	– to evaluate the efficacy of the bulevirtide combination with peg-IFNα in participants with HDV. – the number of participants with sustained virological response at week 24 (undetectable) was also assessed.
REP 2139-Ca Nucleic acid polymers Block viral entry	REP 2139-Ca pegIFNα-2a	12	Interventional	Phase 2/ NCT02233075	– to examine the hypothesis that combined REP 2139-Ca/pegIFNα-2a treatment is safe and well tolerated in patients with HBV-HDV co-infection, and that NAPs are an effective approach for clearing HBsAg from the serum of HBV-infected patients.

## Data Availability

No new data were created or analyzed in this study. Data sharing is not applicable to this article.
